# Responsible innovation in multi-organ-on-a-chip research: A qualitative study of biomedical scientists' views on the value and development of models for obesity

**DOI:** 10.1016/j.namjnl.2026.100134

**Published:** 2026-09-15

**Authors:** Mila I. Rep, Franck L.B. Meijboom, Sterre D.E. Kieft, Manon van Daal, Marianne C. Verhaar, Bas W.M. van Balkom

**Affiliations:** a Department of Nephrology and Hypertension, University Medical Center Utrecht, Utrecht, The Netherlands; b Center for Sustainable Animal Stewardship, Division of Animals in Science and Society, Department of Population, Health Sciences, Faculty of Veterinary Medicine, Utrecht University, Utrecht, The Netherlands; c Department of Bioethics and Health Humanities, Julius Center for Health Sciences and Primary Care, University Medical Center, Utrecht, The Netherlands

**Keywords:** Advanced *in vitro* platforms, In-depth interviews, Experts' insights, Content analysis

## Abstract

Obesity is a global health issue and risk factor for various diseases. Given its systemic nature, multi-organ-on-a-chip (MOC) models have been proposed to model human organ interactions *in vitro.* Obesity MOC models are still in an early stage of development. Early stakeholder engagement may provide insights into societal, scientific, and ethical aspects of obesity MOC models, and incorporating these dimensions can support their development and implementation. Therefore, we conducted an explorative qualitative study focusing on academic and industrial biomedical researchers’ views on MOC models for obesity, based on a hypothetical model incorporating adipose tissue, kidney, and liver organ systems. Analysis of the semi-structured interview transcripts identified six themes that consistently emerged across all participants. Our results show that participants perceived obesity as a global health issue requiring prevention efforts. However, researchers argued that obesity MOC models should mimic human (patho)physiology, rather than focus on prevention. Moreover, tensions between capturing human system complexity and maintaining manageable technical setup were identified. Perceived hurdles, including finding universal culture medium, limited affordability, and inadequate high-throughput capacity, may hamper obesity MOC model development. Clear research objectives and model validity were considered crucial, and explicitly defining context of use may facilitate progress. Divergent views on obesity MOC model validation, ranging from animal replacement to animal benchmarking, highlight tensions in scientific practice. In conclusion, consensus on the value and validation of obesity MOC models requires engagement among scientists, institutions, and society. Our findings can inform design and communication of obesity-related MOC experiments, supporting their development and use.

## Highlights

• This qualitative study explored the views of academic and industrial biomedical researchers on the development of obesity multi-organ-on-a-chip models to shape future biomedical obesity research.
• Although researchers link behavioural and lifestyle factors to obesity and emphasize the importance of preventing obesity, these factors are not included in obesity MOC models' assessments.
• Perceived potential hurdles in the development of obesity MOC models are their affordability, finding common medium for maintaining multiple organ systems, and their suitability for high throughput.
• Future research should draw on broader stakeholder input to ensure that societal and ethical aspects, including conversations about what does and does not count as valid evidence for obesity MOC models, are addressed within the framework of responsible research and innovation.

## 1 Introduction

In obesity, excess body fat accumulates primarily in white adipose tissue (Reyes-Farias et al., 2021). Excessive fat makes individuals with obesity prone to numerous health risks, including cardiovascular, kidney, and liver diseases (Delpino et al., 2023; Guh et al., 2009). By 2035, over 50% of the world’s population will be overweight or living with obesity (WOF, 2024). The rising prevalence of obesity emphasizes the need for research that enables patients, healthcare systems, and societies to better cope with obesity-associated comorbidities and risks. Addressing obesity-related effects requires research models that accurately represent systemic human physiology and reflect inter-organ communication (Juguilon et al., 2025).

Currently, preclinical obesity research relies on two main model types: *in vitro* cell culture models and *in vivo* animal models. Cell culture models, particularly monolayer cultures of primary (pre)adipocytes (Dufau et al., 2021) which are relatively homogeneous and genetically well characterised, or patient derived adipose-derived stromal/stem cells which have high physiological relevance and allow investigation of patient-to-patient variation (Muller et al., 2019). Such cell culture models are cost-effective and suitable for large-scale assays. However, these models fail to replicate the complex physiology of living organisms. Particularly, they do not capture 3D tissue organization and systemic physiological effects (Avelino et al., 2024). Animal models, like obese mice or rats, can partly mimic complex physiological environments and systemic responses (Ghanemi et al., 2021). Nevertheless, animal models are costly, time-consuming, ethically challenging, and limited in terms of their translation value to humans (Frangogiannis, 2022; Rhrissorrakrai et al., 2014). Growing recognition of the ethical and scientific limitations of these research models has brought a shift towards more advanced and human-centred *in vitro* approaches (Suleiman et al., 2019).

An appealing option for advanced and human-centred *in vitro* approaches for studying systemic effects in obesity is the use of multi-organ-on-a-chip (MOC) systems, which are designed to replicate the complex interactions between multiple human organs (Picollet-D’hahan et al., 2021). MOC platforms have gained significant traction for drug development, disease modelling, and personalized medicine (Ma et al., 2021; Sunildutt et al., 2023). The pharmaceutical industry, as a primary end-user, has shown particular interest in using MOC systems to test its products (Vollertsen et al., 2021) and is incorporating MOC systems into their research for safety, efficacy, and toxicity testing (Marx et al., 2020, 2025). However, MOC models for obesity research are in the early stages of development (Picollet-D’hahan et al., 2021).

To use data from MOC models in pharmaceutical regulatory submissions and decision-making, several key phases are required, including standardization, validation, implementation, regulatory approval, and acceptance (National Institute for Public Health and the Environment RIVM, 2024). Beyond the scientific aspects, it is also important to understand societal and ethical concerns to successfully move through these phases. Scientific research is neither value-free nor purely objective, and is embedded in societal and ethical views. In line with Responsible Research and Innovation (RRI) (Von Schomberg, 2013), the evaluation of ethical and societal implications of novel developments involves early stakeholder engagement, which broadens the range of perspectives on what is societally and ethically relevant in the development of novel innovations (Owen and Pansera, 2019; Stilgoe et al., 2013).

To explore the societal and ethical aspects of MOC models for obesity research, we conducted an exploratory qualitative study focusing on the views of academic and industrial researchers on the value and development of obesity MOC models. Qualitative interviews are a valuable method to identify, better understand, and juxtapose people’s perspectives. In this way, qualitative research can improve the understanding of ethical and societal implications of MOC models by providing insights into how interviewees contemplate different ethical and societal aspects (Rehmann-Sutter et al., 2012). We chose these stakeholder groups because of their direct involvement in research on advanced cell culture models or animal models. Our aim was to capture a diversity of views and perspectives, rather than to make representative statements of a specific group of scientists. Based on the research question ‘*How are MOC models mimicking obesity perceived by academic and industrial biomedical research professionals?*’, we mapped relevant themes and key factors in the discussion of the use of MOC models for obesity, from which we identified hurdles and facilitators in the development of such models. With a predicted increase in research on obesity, results from the current study may help shape the research topics, directions, and models in this field. Furthermore, these insights will contribute to the societal relevance, acceptance, and adoption of MOC models in general, and those mimicking obesity specifically.

## 2 Methods

### 2.1 Study design and reporting

This study aimed to explore views on the development of MOC platforms for obesity from 1) academic biomedical researchers and 2) industrial biomedical researchers. Given the explorative character and the aim to search for perceptions of professional stakeholders and the underlying arguments, we used a qualitative approach by conducting qualitative interviews (Creswell and Creswell, 2018). We conducted semi-structured interviews. Interviews were chosen over focus groups and surveys because topics such as obesity and laboratory animal use may be sensitive to talk about. Additionally, semi-structured interviews allowed for a deeper exploration of participants’ views and enabled the interviewer to demonstrate empathy, encouraging more open responses using a post-positivism and critical realism epistemological framework. Post-positivism acknowledges that complete objectivity is not possible, but strives for transparency and systematic methods to minimize subjective biases (Robson, 2002), and critical realism recognizes an inherent subjectivity in knowledge formation, such as the researcher's interpretations (Madill et al., 2000). Therefore, during thematic analysis, the identified themes were interpreted not only as descriptions of participants' experiences but also as indicators of broader organizational and social contexts influencing those experiences.

The present study is reported according to the criteria mentioned in the COREQ guideline (Tong et al., 2007), which are detailed in Supplementary Appendix 1. The first author (MIR) is female, trained as a psychologist (degree: BSc) and biomedical scientist (degree: MSc). MIR is a native Dutch speaker with high proficiency in English.

### 2.2 Participant selection and recruitment

Given the study's aim, *i.e.*, exploring views on the development of MOC models for obesity, we recruited scientists with diverse backgrounds from various work locations across the Netherlands as relevant professional stakeholders to gather feedback on the idea of using MOC models for obesity research. To ensure the pseudonymity of the participants, these specific characteristics are not reported in this paper. To increase the diversity of perspectives, we selected participants along three dimensions. Firstly, we selected interviewees from different career stages (early, mid, and senior). During the interviews, participants indicated which stage applied to them. Secondly, we included scientists with different levels of involvement in animal use in their professional lives. This included a range from scholars who do animal testing to scientists who do research without using animal models. Finally, we selected the interviewees from two target groups: scholars working in academia and in industry (Table 2). We selected academic scientists as they are directly confronted with and involved in addressing ethical and societal concerns about the development and application of emerging technologies such as MOC models, and in the dialogue about the necessity, reduction, and replacement of animal experiments. Scientists from the industry are relevant stakeholders because they connect fundamental research with the development of concrete products, processes, or models that can be directly applied in the market. These scientists are also confronted with the ethical and societal dimensions related to MOC models. Participants had no direct personal or professional relationship with the interviewer. Potential interview candidates were invited via e-mail. In total, fifteen (out of seventeen) scientists agreed to participate. The research protocol was submitted to the research ethics committee of the Utrecht University for review before starting the research. The committee determined that this study was exempt from the Medical Research Involving Humans Act (registration number Bèta S-23,985).

In line with the research protocol, we obtained written informed consent from all participants. Before the interviews, participants received an invitation letter (Supplementary Appendix 2) and the interview guide (Table 1), which explained the study’s background, design, topics, duration, and data management (in line with the General Data Protection Regulation). In the consent form, participants confirmed they were well informed, participated voluntarily, could withdraw, and agreed to have their voices recorded.

**Table 1:** Interview guide.

Topic		Main questions
Demographic and research profession	1. What are the specific activities/responsibilities in your profession? 2. How would you describe a regular workweek? 3. (What kind of research are you currently involved in?) 4. Do you consider yourself in stage of early career, mid-career of senior career? 5. What motivated you to work as [profession they just mentioned] in your field? 6. What is the role of animals in your profession?	
Opinion on obesity and models for investigating obesity	7. What are your thoughts about the current epidemic-like occurrence of obesity? 8. What kind of research would be relevant to study obesity?	
Obesity-on-a-chip model	9. What is the first thing that comes into your mind when hearing this obesity-on-a-chip model? 10. Do you consider such models as relevant for investigating obesity? 11. Do you consider such model as valid for investigating obesity? 12. Do you consider such model as representative for investigating obesity? 13. What could be a challenge in the development of such models? 14. How can we tackle the problems you just mentioned? 15. What would you consider as a success for such models?	
Future perspectives	16. How do you see the future regarding the use of different models to investigate obesity? 17. How close are we towards this future perspective? 18. What needs to be done to reach this future perspective?	
Round-up	19. Are there important aspects about investigating obesity and the different models to study obesity that I have forgotten to ask about? 20. Is there anything else you would like to mention?	

### 2.3 Data collection

Fifteen interviews were conducted from 11 May until 27 June 2023, either in face-to-face meetings or via online video meetings. The interviews lasted approximately 45 min. All interviews were conducted in English by MIR.

The interview guide was divided into five parts (Table 1). For building rapport, we started by asking demographic questions such as specific activities related to the interviewee’s current profession and the role that animals play in their profession. Second, the participants were asked to explain their perspective of the current epidemic-like occurrence of obesity and relevant types of research for investigating obesity. Third, a conceptual human obesity-on-a-chip model was introduced. In short, the interviewer explained the following: This chip of two circuits is a miniature system of chambers and channels in a laboratory setting with a flow of medium. It aims to mimic the function and the behaviour of the three human organ systems, namely fat tissue, kidney, and liver. Fourth, questions regarding the perspective of the participants about the obesity-on-a-chip were asked to go more in-depth about their perspectives on MOC models for obesity. Questions regarding their perspectives on the relevance, representativeness, and validity of such MOC models for investigating obesity were asked. The interviewer provided clarification on these concepts when necessary to ensure a shared understanding of the questions, while allowing participants to articulate their own interpretations. Although some questions were phrased as yes/no prompts, they primarily served as starting points for discussion and were followed by open-ended probing questions to further explore participants’ perspectives. Furthermore, this section included questions about what the interviewee considered to be challenging in the development of obesity MOC models, how such challenges can be addressed, and when one would consider such models to be successful. In the fifth and final part of the interview, questions focused on their future vision on the use of different models to investigate obesity were asked, and how this needs to be reached. During the interview, the researcher summarized information for corrections and additions and asked occasional follow-up questions.

Notes and voice recordings were taken during the interviews. After this, the interviews were transcribed verbatim using audio transcription via Microsoft Word (Microsoft Inc.) and manually edited to correct any transcription errors. The transcripts were pseudonymized, which means the files were coded with a number to ensure pseudonymization and objectivity. This allowed MIR, FLBM and SDEK, the researchers, to perform the data analysis in a blinded manner, without knowledge of the participants’ identities.

### 2.4 Data analysis

To analyze the data, we followed a step-by-step analysis plan (Verhoeven, 2023), based on six phases of Braun and Clarke (Braun and Clarke, 2006): exploring, coding, thematizing, revising and refining, determining and structuring, and presenting. We used an inductive and semantic form of reflexive thematic analysis (TA) on the interview data (Braun and Clarke, 2006, 2021a). This approach means that themes can be developed by finding shared patterns of meaning within the participants’ responses. ‘Meaning’ at a semantic level is considered to be explicit within the language of the participants.

Analysis began with repeated reading of the transcripts to achieve familiarity with the data. Relevant data were coded using descriptive labels, and codes were iteratively refined and consolidated. Coding proceeded iteratively until assessors MIR, SDEK, and FLBM judged the dataset to be sufficiently rich and comprehensive to support a robust interpretation of participants' perspectives in relation to the research question (Braun and Clarke, 2021b). Codes were then grouped according to conceptual similarities and organized into higher-order concepts, from which candidate themes were developed. Through an iterative process of review and refinement, final themes were generated that addressed the research question (Hennink and Kaiser, 2022). Data were managed and analysed using NVivo 15 (QSR International) without AI assistant. In the Result section, scientists have assigned codes per sector (A for academic scientists and I for industrial scientists) and a number.

## 3 Results

Participants represented different career stages, professions, and the use of cell culture and animal models in their research (Table 2A, Table 2B). The analysis of the transcribed interview data has led us to identify six overarching core themes, which consistently emerged across all interviewees, regardless of career stage, profession, or current use of animal models: (1) societal and scientific context of obesity research; (2) scientific aim, (3) biological complexity, (4) technical setup, (5) validation, and (6) comparison with *in vivo* animal models (Fig. 1). After discussing Theme 1, Theme 2 to 6 emerged from the researchers’ discussions around the development of MOC models for obesity, using the adipose tissue–kidney–liver model as an example.

**Table 2A:** Overview of career stage of the interviewees.

Stakeholder group /Career stage	Early	Mid	Senior
Academic scientists	4	4	2
Industrial scientists	0	3	2

**Table 2B:** Overview of interviewees’ involvement in research with animal models.

Stakeholder group /Animal use	Yes	No
Academic scientists	5	5
Industrial scientists	1	4

**Fig. 1:**
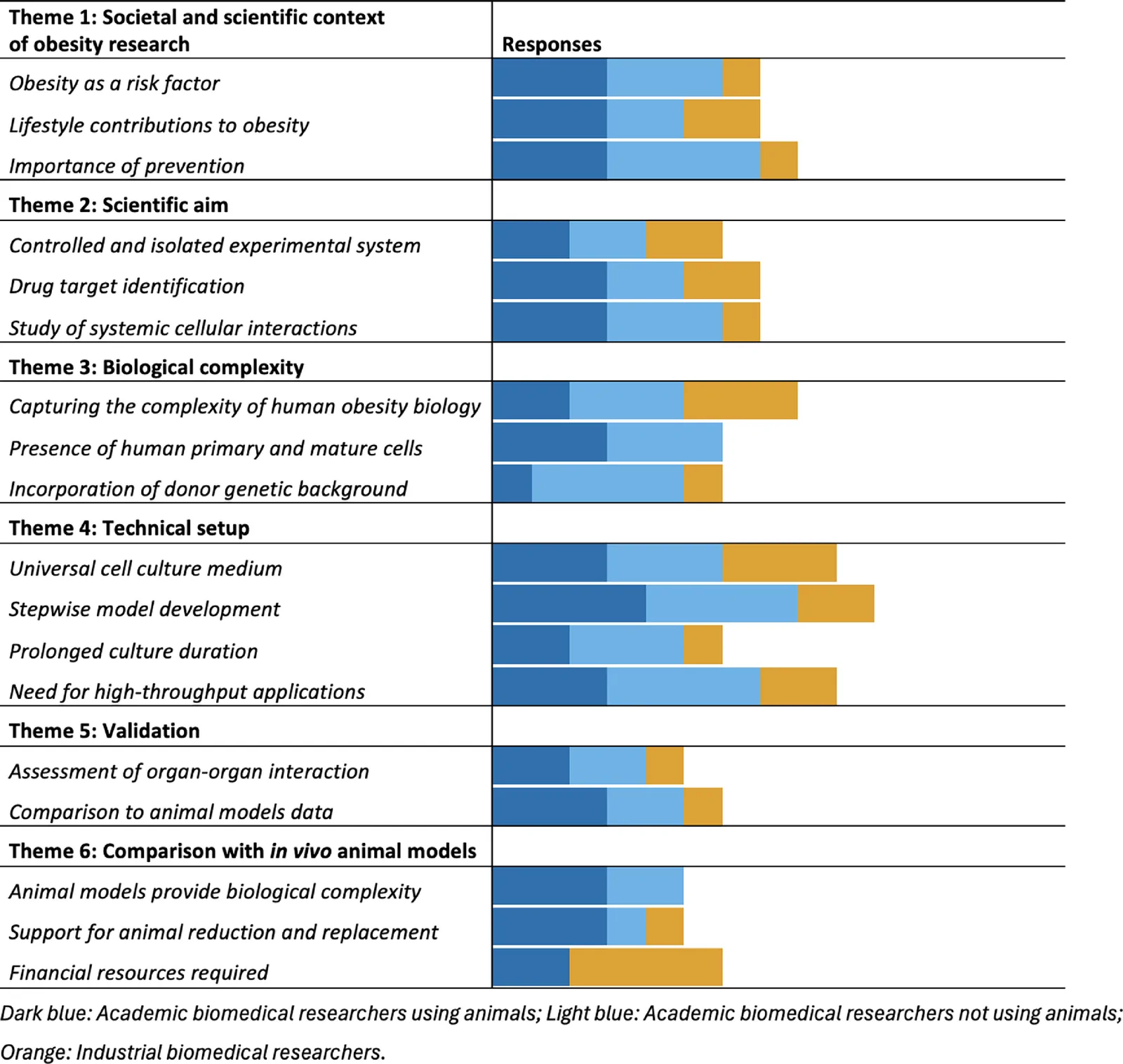
Overview of main responses of the interviewees.

### 3.1 Theme 1: societal and scientific context of obesity research

The first overarching theme that emerged from the data is the societal and scientific context of obesity research. The interviewees viewed the epidemic-like occurrence of obesity as sad or worrisome (I1, A7) and as a significant and increasing problem (A1,4–9). They perceived obesity as a societal (A1,6, I5) and international (A6–8) problem. The negative effect on general health, *e.g.*, the link of obesity to various other diseases, was highlighted (A1,2,4,7–9, I4). Furthermore, participants mentioned the economic impact on the healthcare system (A2,3, I1,3).

Moreover, participants discussed several causes of the epidemic-like occurrence of obesity. Most scientists indicated that obesity is driven by lifestyle (A2,4,5,7,10, I1,3), by the availability of food (I4, A6), or mental health problems (A9). This lifestyle dimension, according to one interviewee, was a reason that the biology of obesity is still insufficiently investigated (A8). Furthermore, it once sparked a discussion with a participant about who is responsible for this problem: “*Are obese people the ones to blame?”* (A8).

When considering ways to tackle the increasing occurrence of obesity, the interviewees stressed the importance of prevention (A1,2,4,6,8–10, I4). Ways of prevention included ideas on educating people (A3), creating awareness (A6,7), changing the supply chain and marketing, and stimulating exercise (A6). In addition to these preventive strategies, the importance of investigating the social and societal aspects of obesity was highlighted (A2,5–7, I1,3–5). One participant suggested that a focus on biomedical research may not help society and argued that food affordability should be improved, and the prevalence of fast-food restaurants should be reduced (A5). Others mentioned the relevance of research into the effect of fat mass on bones under gravity and chronic inflammation (A1), pathology (A2,7,9, I5), biology of obesity (A1,8, I3), decision-making processes in the brain (A9), human cells (I2). Also, studies into the role of relevant genes were suggested (A5,6,9, I1,5), whereas others questioned such research (A4,9). One interviewee brought up the relevance of research into the pharmacology of people with obesity since drug effects may differ substantially, given that most medications are developed based on average body characteristics (A6). Also, some participants pointed out the importance of investigating practices within the food industry (A5), human dietary patterns (A6,9), and the availability of food (I3).

### 3.2 Theme 2: scientific aim

A second overarching theme is the scientific aim of MOC models for obesity. Several scientists stated that the ultimate goal of MOC models for obesity is to mimic the human *in vivo* situation (A2,4,7,8, I1). Two scientists claimed that the obesity MOC model only needs to resemble a specific part of obesity or an organ, not the full *in vivo* situation (A9, I3). In line with this more narrowly defined goal, some participants mentioned that MOC platforms are recognized as providing a controlled setting to study isolated processes, allowing specific mechanisms to be examined without interference from the rest of the system (A1,5,7,10, I4,5). Participants noted that it should be considered that the envisioned obesity-on-a-chip primarily mimics the influence of secreted factors on cellular systems (A3,7,10, I1,4). Some participants emphasized that these cellular systems mimic biological processes at the cellular rather than the tissue level (A1,2). Some stressed that the model has non-natural environments for the cells, raising concerns that findings may lack physiological or clinical relevance (A4, I3,4).

It was discussed that once the aim of (partially) mimicking the human *in vivo* situation is achieved, MOC models can be used for various purposes, such as finding important human secreted biomarkers (A4), targets for drugs (A1,3,5–7, I1,5), and investigating cellular interactions between different human organs (A1,2,4,6,8,9, I1). For specifically mimicking obesity in MOC models, scientists questioned if the obese phenotype was created by a higher number of adipocytes, by increased fat accumulation (A4,8,9), or changes in diet (I5). One participant asked: “*Can you push the model in a pathological direction? (…) The border of obesity is complex.*” (A6). One interviewee noted that fat accumulation is difficult to replicate *in vitro* 2D cell models (I2).

Furthermore, interviewees discussed the importance of clear research objectives for MOC models for obesity to assess their potential impact (A3,8–10, I4). “Y*ou should centralize the question you prefer to answer. (….) Look at the benefits of your system versus the potential flaws of the system.*” (A9).

### 3.3 Theme 3: biological complexity

Given the multifactorial character of obesity (theme 1) and the aim of mimicking the human situation (theme 2), complexity also emerged as a third theme. Participants evaluated to what extent the MOC models for obesity capture the complexity of human obesity biology (A1,4,6,8,10, I3–5). Some considered such models a simplification of the human system (A6,10, I3–5) since “*it [hypothetical obesity MOC model] will only be able to model relatively simple communications between cells.*” (I3). One scientist noted that simplified models may overlook complex cell interactions present in the human system (A8). Some participants raised a question about the level of complexity required to accurately represent *in vivo* processes in general (A4,8). One scientist said it is challenging to prove the model is not oversimplified (I5).

With the goal of mimicking the systemic part of obesity, the combination of multiple organ systems was seen as positive (A4, I2,3). Several cell features were mentioned as important, such as human (A4,5,9,10), primary origin (A2), and maturity (A4,10). One participant proposed using cells from the same donor to generate all three organ systems, but also emphasized the difficulty of obtaining cells from multiple organs from a single donor (I1). To overcome these problems, some interviewees proposed the use of induced Pluripotent Stem Cells (I1, A5). One participant emphasized that selecting the appropriate fat depot for cell collection is crucial (A3). Moreover, several participants highlighted the importance of considering the genetic backgrounds of individuals who serve as cell donors (A3,6,8–10, I2). Some participants also mentioned that obtaining cells from different donors for every component or different diseases might overcomplicate the models (A6) and is difficult (A6, I5).

To increase the level of translatability towards the *in vivo* human situation, most interviewees suggested adding other biological components in the MOC model, for example, components to model inflammatory responses (A1,7, I1), endothelium or its extracellular matrix (A2,4,5,7, I4), the lymphatic system (A2), and hormone dynamics (A6).

### 3.4 Theme 4: technical setup

Building on discussions of MOC model biological complexity (theme 3), participants also highlighted the technical requirements needed to achieve and maintain such complexity. These considerations formed a separate fourth overarching theme focused on the importance of the technical setup. Participants noted that increasing the technical setup of MOC models for obesity affects the ability to assess causal effects (A3–5,7–10). Some scientists advised not to further increase the complexity of the MOC model for obesity beyond a three-organ model (A2,9). To study causal relationships, it was suggested that organ systems can be introduced incrementally, adding one organ system at a time (A7,8,10). In line with this thought, interviewees mentioned that the development of MOC models for obesity is a step-by-step process (A1,3–9, I2,4)

Given the notion that MOC models primarily mimic the influence of secreted factors on cellular systems, scientists questioned the need to integrate these systems within a single model and proposed using conditioned medium from separate cell cultures instead (A6,9, I3,4). One researcher noted that models are often made unnecessarily complex to be considered publishable (A3).

Interviewees mentioned technical challenges that could impact the development and implementation of MOC models. Firstly, participants emphasized the need for a common medium to maintain multiple cell types in a combined MOC system (A1,2,5–7,10, I2,4,5). One scientist added: “*It is important to consider how often, how much, and at what time points you refresh.*” (I2). Also, participants mentioned the importance of considering batch-to-batch differences in three-dimensional hydrogels, such as Matrigel and Basement Membrane Extract, in which organoids are usually cultured (I1, A5). In addition, several interviewees expressed the challenge of prolonged cell culture time (A1,5,6,9,10, I3). One scientist added to this challenge: “*Making an organ takes time. Creating organoids in a couple of weeks is already amazing*. (…) *Accept that it [cell culture] takes time.*” (A6).

Other technical considerations of MOC platforms were mentioned, such as flow (A1,3,4, I3,4) and air bubbles (A10). One respondent said company-made chips are more reliable than chips made by academic research groups (A7). Also, the need for high-throughput (A1,4–7,9,10, I1,2) approaches for MOC models was mentioned, which might be difficult to achieve due to the complexity of the model (I2) or the high costs (A6). The described complexity and resulting relatively low throughput make it difficult to find the true effect, according to another scientist (I2). Some suggested that throughput can be increased by industrial adoption (A7, I1) since “*industry has automated microscopes (……) and robots for cell culture.”* (I1). Some participants noted that cost considerations play a role in decisions to use such MOC models (I2, A3).

### 3.5 Theme 5: validation

The fifth overarching theme that emerged from the data is validation. Considering the objective of mimicking the human *in vivo* situation (theme 2) and the technical setup (theme 4), scientists highlighted that validation of MOC models can be achieved by manipulating one of the organ systems to observe an effect in another (A1,2,8,9, I4). They stressed that the specific design of such an experiment depends on your research question (A1,2). Often, it was suggested that the findings obtained in MOC experiments can be validated against data from studies with humans (A4,7), humans that are treated (A2, I1), *in silico* models (A3), or animals (A2,4,5,7,8, I4), such as a mouse model “*with low-grade chronic inflammation and the same metabolism as humans*” (A2). One participant mentioned that benchmarking against animal data may also be important for publication in high-impact journals, as it is considered that reviewers would like to see whether the mechanisms in MOC are acting similarly *in vivo*. “*They [reviewers] think it is closer to the patient than in vitro”* (A10).

One scientist highlighted benchmarking challenges, including the consideration that effects observed in MOCs may exceed those seen in treatment: “*Because you isolate, you also magnify*.” (A1). Also, some participants cautioned that validation can be hindered by the limited informational yield of these MOC systems (A3,7). For example, one participant mentioned that the low number of cells makes it challenging to obtain RNA and perform gene expression analysis (A3). Some scientists emphasized that MOC models must outperform existing models significantly to gain acceptance (A1,5).

### 3.6 Theme 6: comparison with *in vivo* animal models

The final overarching theme following the data is the need for comparison with *in vivo* animal models, especially in the light of discussions around the aim of mimicking the human situation (theme 2) and the use of animal models for validation (theme 5). Several interviewees emphasized the need for animal models due to their biological complexity (A1–3,5, I3,4), their (past) relevance for understanding human biology (I3), and the presence of a vascular system (A10). One scientist considered animals as a simplification of the human system (A10), and others viewed them as systems in which inputs generate outputs without fully clarifying the underlying biological processes (A1,5, I4). Genetically obese animals, intended to mimic human obesity, were not considered suitable for studying obesity causes, but were useful for studying its consequences, according to one interviewee (A4). Some scientists said animal models allow the use of specific diets to induce obesity (A2,4), whereas another scientist argued that obesity cannot be adequately modelled in animals through overeating (A1).

Participants noted that animal reduction and replacement are beneficial in general (A3–5,7, I3), but will not be achieved soon (A1,4,5,7, I3). Some scientists expressed the potential of MOC models to reduce or replace animals (A1,5,7). However, others suggested that animal models will be preferable (I3,4) since “*Animal models are still more similar to human beings than culturing cell types together in this chip*” (I3). Some participants suggested that MOC models can serve as an additional model next to animal models, so it helps to reduce animal models (I2,4,5), for example, “*to verify your hypothesis*” (I4). One scientist emphasized that “*we should not oversell the hype. (…) Animal replacement should not be the aim, but the result*” (A1).

Many participants stressed that collaboration between academia, companies, and the government is essential to achieve the replacement of animal models (A4,5,7, I1). Some participants addressed the need for financial resources (A5,7) as MOC models and cell culture medium are costly (A1,2,6, I1,2,5). One researcher added that this makes it “*costly for pharma to step into this [using MOC models]*“ (I1). One scientist emphasized the interest from industry in MOC models and highlighted differences between industry and academia: “*The industry keeps an eye on all models. If there is urgency, we choose. (…) It depends on whether we can make money. Academia has research lines for the next twenty years, whereas we have two or three years of development.”* (I2).

## 4 Discussion

Our explorative qualitative study revealed researchers’ perspectives on the development of obesity MOC models. Our findings show that obesity is acknowledged as a significant public health problem with a complex and multifaceted nature, lacking a single, easy solution. Research in social and public health domains was highlighted as crucial for addressing problems of obesity, consistent with prior research (Bombak, 2014; Młynarska et al., 2025; Singh et al., 2025; Wang et al., 2015). Scientists linked behavioural and lifestyle traits to the development of obesity and emphasized the importance of obesity prevention. This contrasts with the limited attention given to sociocultural and psychological dimensions and prevention of obesity when assessing obesity MOC models. Researchers proposed mimicking (parts of) human physiology in obesity MOC models. To mimic parts of human physiology, integration of biological complexity in obesity MOC models was a central discussion point. Concerns regarding the artificial nature of the cellular environment, the selective use of specific organ systems, and the absence of other biological components raised questions about which elements are necessary to accurately mimic human physiology. These concerns relate to how uncertainty in obesity MOC models should be addressed, particularly with respect to internal and external validity, and to what extent such uncertainty is acceptable, especially in light of the ethical responsibility to ensure that findings do not misinform subsequent research, including pharmaceutical development and other translational efforts. These challenges highlight the need for careful characterization, evaluation, and communication of scientific uncertainty to ensure that decisions in science are neither over-trusted nor under-trusted (Fischhoff and Davis, 2014; Van Der Bles et al., 2020).

In our study, there was a tendency to view MOC model development from a biomedical and technical perspective. This emphasis on biological, physiological, and technical aspects, rather than sociocultural and psychological ones, has also been observed in other reports evaluating MOC models (Juguilon et al., 2025; Leung et al., 2022). It may reflect scientists’ everyday research practices, in which they focus on evaluating the physiological relevance and optimizing the cell and animal models used in their research. This attention may be associated with a stronger focus on technical and biological concerns, potentially at the expense of ethical and societal considerations. It could also be attributed to the formulation of the interview questions with terms such as representativeness, relevance, and validity, which might have steered them towards assessing the MOC model by its biomedical and technical components. Another explanation would be the historical boundary between biomedical and social science research. This boundary work sustains a division of labour where laboratory research is expected to remain “purely biological,” leaving social determinants to epidemiologists, sociologists, or public health scholars (Adam, 2011).

It is important that this division of labour is questioned and, where necessary, adapted, especially in the biomedical field. Emerging biomedical innovations are paired with ethical and societal implications (Jongsma and Bredenoord, 2020). In the context of MOC models, discussions include the use of human tissues. For instance, potential donors for organoid research felt a personal connection to organoids and have described them as ‘living human materials or living fragments of themselves’ (Boers et al., 2018; De Jongh et al., 2022; Haselager et al., 2020). A reason for this perception was the 3D structure of organoids, which sets them apart from other immortalized cells (De Jongh et al., 2022). This requires researchers to exercise additional awareness while handling such tissues in the lab and ensuring stronger safeguards for privacy. Both academic and industrial biomedical researchers must be aware of these ethical and social dimensions, so they can account for them in their daily practices. This requires an interdisciplinary approach in developing MOC models for obesity.

The researchers did not express principled objections against the development of obesity MOC models and viewed it as part of a stepwise progression in research. Their comments illustrate that this progression entails multiple facilitators and hurdles in developing MOC models for obesity. Although a concise and systematic overview of facilitators and hurdles cannot be directly derived from our data, three perceived hurdles were commonly reported, namely affordability, high-throughput requirement, and the necessity of a universal medium to combine multiple cell organ systems, which have been widely recognized in the field (Alver et al., 2024; Olaizola-Rodrigo et al., 2025; Probst et al., 2018). Researchers have proposed various applications for the obesity MOC model in basic research, clinical practice, and drug development. In light of the need to establish clear research objectives and ensure model validity, defining the context of use may serve as an important facilitator. Here, context of use refers to the specific purpose or application for which the model is intended, and the conditions under which it is meant to be applied. This is crucial for evaluating its relevance, reliability, and regulatory acceptability. Previous papers mainly frame the context of use in biomedical and technical terms, with societal and ethical dimensions only rarely made explicit (Hickford et al., 2023; Stokar-Regenscheit et al., 2024). For obesity MOC models, both behavioural and physiological dimensions should be considered, given the complex interaction between these dimensions. Obesity is strongly influenced by behavioural factors in its development and progression. To define an appropriate context of use for obesity MOC models that addresses technical, ethical, and societal concerns, collaboration is needed among stakeholders such as regulators, funders, and pharmaceutical companies. This aligns with a recent workshop report on biology-inspired models, including MOC models, which emphasizes structured multi-stakeholder dialogue for establishing MOC contexts of use that address technical validation alongside ethical and societal (Marx et al., 2025).

Notably, differences were observed in how MOC and animal models were valued. While some researchers highlighted obesity MOC models as potential alternatives to animal models, others emphasized validating MOC-derived results against animal data, given their biological complexity. The latter may reinforce the perceived authority of animal experiments, raising questions about how scientific validity is socially constructed and legitimized. Given fundamental interspecies differences and the limited capacity of animal models to predict outcomes in complex biological systems such as obesity, these models may be insufficient on their own, whereas MOC models could offer advantages by better capturing human-relevant complexity (Lempesis et al., 2022; McCarthy et al., 2020).

There are many reasons why animal models remain highly valued in biomedical research. Their use is deeply embedded within academic institutions and industrial companies. Also, many scientists have built their careers around animal experimentation, which can be difficult to change and can create a personal and emotional investment in maintaining these practices (Greek, 2014). Moreover, the general public often lacks sufficient scientific and medical knowledge to critically assess the limitations of animal models *versus* other models. Finally, ethical debates on animal research often focus on the pain and suffering of animals, whereas the potential consequences for human health and outcomes should also be an integral part of these discussions (Greek, 2014). A shift is needed in what counts as valid evidence and how non-animal models are valued, which can be done through debate, controversy, consensus-building, and “closure” within a community (Fernández et al., 2022; Parvatam et al., 2024). Therefore, interactions among scientists, institutions, and broader societal actors are needed to establish a new consensus on the value of, and the requirements for validation of, obesity MOC models.

## 5 Limitations

It should be taken into account that our study may have certain limitations. Participants might have given socially desirable rather than fully honest answers because of perceived social norms or expectations within the interview context (Bispo Júnior, 2022), especially when discussing sensitive topics such as animal experimentation and obesity. To address this potential limitation, we established common ground with interviewees at the beginning of the interview, and emphasized that there were no right or wrong answers (building rapport) (Kypri et al., 2016). Additionally, questions on future perspectives lacked sufficient specificity, leading to broad responses and challenges in proper data analysis. Moreover, participants frequently focused on the envisioned adipose tissue–kidney–liver MOC model rather than MOC models for obesity in general, even with repeated clarification by the interviewer to address the broader context. As a result, the operational definition used in this study may have narrowed the scope of participants' reflections on physiological and pathological representativeness, potentially limiting discussion of other relevant components involved in obesity. Also, as the study was conducted exclusively with participants based in the Netherlands, the findings may reflect views shaped by the Dutch research and regulatory landscape, including ongoing efforts to promote alternatives to animal testing. Consequently, views regarding funding priorities, and developmental challenges, implementation barriers of obesity MOC models may differ from those in other national or international contexts. Furthermore, although the participant cohort included academic and industrial biomedical researchers, representation from industry was relatively limited, and industrial scientists using animal models, and early-career industrial scientists were not represented in the sample. Although perspectives on obesity MOC models’ development were broadly consistent across different career stages and professions, future studies including a broader international sample and more diverse industry representation could provide additional insights into stakeholder priorities and challenges related to obesity MOC development and implementation.

## 6 Conclusion

This article explored how academic and industrial biomedical researchers view factors that influence the development of MOC models for obesity. The findings indicate that challenges associated with obesity are not primarily biomedical in nature. Although the societal context of obesity is acknowledged, it is not yet integrated into discussions on the development of MOC models. These societal considerations can be taken into account by including other stakeholders, such as patients and the general public (Janssens et al., 2024; Woolley et al., 2016), in research on obesity MOC models. Future discussions should focus on how to how to capture the biological complexity of the human system and fulfil the requirements of affordability, high throughput, universal medium, and proper validation in order to reach more advanced technology readiness levels required for broader adoption. Consistent with the principles of RRI (Owen and Pansera, 2019) and in line with the work of Hinterberger and Bea, 2023 and Sample et al., 2019, we consider critical reflection on ethical and societal challenges as an essential building block to strengthen research with advanced human *in vitro* models, such as obesity MOC models. A key aspect of this reflection is fostering meaningful interdisciplinary collaboration between biomedical researchers and scholars in the social sciences and humanities. It also involves critically examining the validity and value of non-animal and animal models. All in all, this study highlights the importance of addressing the ethical and societal context of obesity MOC models and offers insights to improve their design, strategic positioning, and implementation.

## AI statement

During the preparation of this work, the author(s) used Microsoft Copilot, Perplexity AI, and Grammarly for assistance with brainstorming, explaining concepts, grammar checking, and improving sentence formulation. The author(s) reviewed and edited the output as needed and take full responsibility for the content of the published article.

## Funding

This research was funded by the HOMO CRASSUS in vitro project by UMC Utrecht and Health∼Holland, Top Sector Life Sciences & Health, and by the BIQMIMETICS project by the Association of Collaborating Health Foundations (SGF) and by Health∼Holland (grant number TKI-LSHT2019-LSHM20046-SGF), ZonMW, the Dutch Kidney Foundation (grant number 19OP+007), the Dutch Society for the Replacement of Animal Testing (Stichting Proefdiervrij), Stichting Universitas and Astrazeneca AB.

## Ethics declaration

Written informed consent to take part in the study and to publish the article has been obtained from all participants or their legal representatives. The privacy rights of participants have been observed. This study was performed in compliance with relevant laws, regulatory frameworks and guidelines where the research took place. This study was conducted in accordance with Medical Research Involving Humans Act. The Utrecht University granted an exemption. The authors provided the following explanation: No medical intervention

## CRediT authorship contribution statement

**Mila I. Rep:** Writing – review & editing, Writing – original draft, Software, Project administration, Methodology, Investigation, Formal analysis, Data curation. **Franck L.B. Meijboom:** Writing – review & editing, Validation, Supervision, Methodology, Conceptualization. **Sterre D.E. Kieft:** Validation, Software, Formal analysis. **Manon van Daal:** Writing – review & editing. **Marianne C. Verhaar:** Writing – review & editing, Supervision. **Bas W.M. van Balkom:** Writing – review & editing, Validation, Supervision, Project administration, Methodology, Funding acquisition, Conceptualization.

## Declaration of competing interest

The authors declare no competing interests.

## Data Availability

Data will be made available on request.
