# Supplementary material for: Responsible innovation in multi-organ-on-a-chip research: A qualitative study of biomedical scientists' views on the value and development of models for obesity
Source: NAM J. 2026 Sep 15;2:100134. doi: 10.1016/j.namjnl.2026.100134 (PMC13657160; doi:10.1016/j.namjnl.2026.100134)
Supplement: Supplementary file 1 [file mmc1.docx]

COREQ checklist – criteria for reporting qualitative studies (adapted from Tong et al. 2007)

| Domain 1: research team and reflexivity | | |
| --- | --- | --- |
| Personal characteristics | | |
| 1. Interviewer | Which author conducted the interviews? | The first author (MIR) conducted the interviews. |
| 2. Credentials | What were the researcher’s credentials? | MSc student |
| 3. Occupation | What was their occupation at the time of the study? | MIR was employed as a master student at the Department of Nephrology and Hypertension at UMC Utrecht and the Department Population Health Sciences at Utrecht University. |
| 4. Gender | Was the researcher male or female? | MIR is female. |
| 5. Experience and training | What experience or training did the researcher have? | MIR had 1 year of experience in quantitative research because of her bachelor's degree in Psychology and her master's specialization in science communication. |
| Relationship with participants | | |
| 6. Relationship established | Was a relationship established prior to study commencement? | MIR only knew one participant out of fifteen participants personally and worked with this person during a previous internship. MIR did not know the others and had not worked with them before. |
| 7. Participant's knowledge of the interviewer | What did the participants know about the researcher? | Participants knew that MIR is a junior researcher affiliated with UMC Utrecht & Utrecht University, interested in obesity-on-a-chip models and the scientific, practical, and societal aspects around this topic. |
| 8. Interviewer characteristics | What characteristics were reported about the interviewer? | Participants were told that MIR was a junior researcher under the supervision of Bas van Balkom and Franck Meijboom. |
| Domain 2: study design | | |
| Theoretical framework | | |
| 9. Methodological orientation and theory | What methodological orientation was stated to underpin the study? | Reflexive thematic analysis (TA) with an inductive and semantic approach. |
| Participant selection | | |
| 10. Sampling | How were participants selected? | Convenience/ Purposive sampling |
| 11. Method of approach | How were participants approached? | Participants were invited via e-mail. |
| 12. Sample size | How many participants were in the study? | 15 scientists from either academia or industry participated in the study. |
| 13. Non-participation | How many people refused to participate or dropped out? Reasons? | 2 scientists did not respond to the e-mail invitation. |
| Setting |  |  |
| 14. Setting of data collection | Where was the data collected? | Data collection took place online, via MS Teams; participants joined either from home or their office. |
| 15. Presence of non-participants | Was anyone else present besides the participants and researchers? | No. |
| 16. Description of sample | What are the important characteristics of the sample? | 10 academic scientists from NL: 4 mentioned early career as career stage, 4 mid-career, and 2 senior career.  5 industrial scientists from NL: 0 mentioned early career as career stage, 3 mid-career, and 2 senior career. |
| Data collection | | |
| 17. Interview guide | Were questions, prompts, guides provided by the authors? Was it pilot tested? | The interview guide is published as Supplementary Appendix 4. It was tested with three colleagues (one in industry and two in academia). |
| 18. Repeat interviews | Were repeat interviews carried out? If yes, how many? | No. |
| 19. Audio/visual recording | Did the research use audio or visual recording to collect the data? | 15 interviews were audio recorded and notes were taken during the interview. |
| 20. Field notes | Were field notes made during and/or after the interview? | Yes. |
| 21. Duration | What was the duration of the interviews? | The duration of the interviews ranged between 27 and 58 min. |
| 22. Data saturation | Was data saturation discussed? | Data saturation was reached when no new codes or themes emerged. |
| 23. Transcripts returned | Were transcripts returned to participants for comment and/or correction? | No. |
| Domain 3: analysis and findings | | |
| Data analysis | | |
| 24. Number of data coders | How many data coders coded the data? | MIR coded all data, and Sterre coded all data except for the answers to the question: What kind of research would be relevant to study obesity? |
| 25. Description of the coding tree | Did authors provide a description of the coding tree? | No. |
| 26. Derivation of themes | Were themes identified in advance or derived from the data? | In line with the reflexive TA approach, themes were derived from the data. |
| 27. Software | What software, if applicable, was used to manage the data? | Data were analyzed and managed with NVivo version 15. |
| 28. Participant checking | Did participants provide feedback on the findings? | No. |
| Reporting | | |
| 29. Quotations presented | Were participant quotations presented to illustrate the themes/ findings? Was each quotation identified? | Quotations of participants were presented; each of them was identified and pseudonymized (A1-10, I1-5) |
| 30. Data and findings consistent | Was there consistency between the data presented and the findings? | Yes. |
| 31. Clarity of major themes | Were major themes clearly presented in the findings? | Yes, there are six major themes, named and summarized at the beginning of the results section. |
| 32. Clarity of minor themes | Is there a description of diverse cases or a discussion of minor themes? | We do not have minor themes. |

**Reference**

Tong, Allison, Peter Sainsbury, and Jonathan Craig. 2007. Consolidated criteria for reporting qualitative research (COREQ): A 32-item checklist for interviews and focus groups. *International Journal for Quality in Health Care* 19: 349–357. https://doi.org/10.1093/intqhc/mzm042.
