# Supplementary material for: Responsible innovation in multi-organ-on-a-chip research: A qualitative study of biomedical scientists' views on the value and development of models for obesity
Source: NAM J. 2026 Sep 15;2:100134. doi: 10.1016/j.namjnl.2026.100134 (PMC13657160; doi:10.1016/j.namjnl.2026.100134)
Supplement: Supplementary file 2 [file mmc2.docx]

**Research information participant sheet**

# Scientific, practical and societal aspects in developing obesity-on-a-chip models

Mila Rep, Junior researcher at UMC Utrecht & Universiteit van Utrecht

Version date: May 5, 2023

**What is the purpose of this study?**

The purpose of this study is to gain insight into the opinion about obesity-on-a-chip models of scientist from academia and industry. This will be achieved by doing interviews about research on obesity, challenges and successes regarding models for investigation of obesity and future perspectives.

**What will I do if I choose to be in this study?**

If you decide to participate in this study, you can email m.i.rep@umcutrecht.nl so she can contact you for making an appointment. Participation is voluntary and you can stop at any point without explanation.

**How long will I be in the study?**

This interview will take approximately one hour online or at your preferred physical location.

**What are the possible risks or discomforts?**

Since talking about the use of different models for investigating obesity might be a sensitive topic, slightly discomfort can occur. There are no right or wrong answers. If you feel uncomfortable, you do not need to answer certain questions and you can stop this interview at any point.

**Are there any potential benefits?**

The questions of the interview will help to reflect on your thoughts and arguments. It is about questioning, in a positive way, what you think and why you think. Reflection is important to gain the most from your learning.

**Will information about me and my participation be kept confidential?**

Your responses will be kept strictly confidential, and digital data and audio recordings will be stored in secure computer files. Audio recordings will be deleted after transcription. Transcripts will be stored for a maximum of 5 years. Any publications based on this research will not include your name or any other individual information by which you could be identified. The project's research records may be reviewed by departments at Utrecht University responsible for regulatory and research oversight.

**What are my rights if I take part in this study?**

Your participation in this study is voluntary. You will not be paid for your participation. You may choose not to participate or, if you agree to participate, you can withdraw your participation at any time without being penalized. This project is approved by the Ethics Review Board of University of Utrecht.

**Who can I contact if I have questions about the study?**

If you have questions, comments or concerns about this research project, you can talk to one of the researchers. Please contact Mila Rep or Bas van Balkom or Franck Meijboom. If you have questions or concerns about your privacy while taking part in the study, please send an email to: privacy@uu.nl.
